# Pyoderma gangrenosum after deep inferior epigastric perforator flap: How prompt diagnosis and treatment can avoid surgical debridement and further morbidity

**DOI:** 10.1016/j.jpra.2022.05.009

**Published:** 2022-05-16

**Authors:** Joao Bombardelli, Janak Parikh, Suat Morkuzu, Aldona J. Spiegel

**Affiliations:** a Department of Surgery, Houston Methodist Hospital, Weill Cornell Medicine, Houston, TX, United States; b Department of Surgery, Division of Plastic Surgery - Institute for Reconstructive Surgery, Houston Methodist Hospital, Weill Cornell Medicine, Houston, TX, United States

**Keywords:** Pyoderma gangrenosum, Deep inferior epigastric perforator flap, Breast reconstruction

## Abstract

Pyoderma gangrenosum (PG) is a skin disorder characterized by painful, enlarging necrotic ulcers with bluish borders surrounded by advancing zones of erythema. The key histologic feature is neutrophilic infiltration of the superficial and deep layers of the dermis and the absence of microorganisms. Although rare and associated with autoimmune diseases such as rheumatoid arthritis, ulcerative colitis and Crohn's disease, the diagnosis is commonly missed at presentation and patients are often treated for infection with antibiotics and surgical debridement. We present a case of PG in a 51 year-old woman after a deep inferior epigastric perforator (DIEP) flap for breast reconstruction who was promptly diagnosed and treated with steroids with appropriate response. Our case highlights the importance of rapid diagnosis and treatment of this disease to avoid incorrect management including surgical debridement, which can exacerbate the disease and increase its morbidity.

## Introduction

Pyoderma gangrenosum (PG) is an inflammatory, noninfectious, ulcerative neutrophilic skin disease.^1^ The disease was first described by Brunsting et al. in 1930.^2^ It is characterized by painful, enlarging necrotic ulcers with bluish undermined borders surrounded by advancing zones of erythema.^1^^,^^2^^,^^3^ The two primary variants are a classic ulcerative form, which often involves the lower extremities, and a vesiculobullous form, which is more superficial and tends to occur on the upper extremities. Skin pain is a prominent symptom. In about 50% of cases, there is an association between PG and systemic diseases such as ulcerative colitis, Crohn's disease, arthritis, myeloma, and leukemia. Surgical intervention is a common exacerbating factor because PG demonstrates pathergy, a phenomenon by which skin trauma can lead to worsening disease.^4^^,^^5^^,^^6^

We describe a case report of PG following bilateral breast reconstruction with a deep inferior epigastric perforator (DIEP) flap. The case highlights how important the early diagnosis and treatment of this condition is to avoid unnecessary debridement that can cause significant morbidity to the patient.

## Case report

**Fig. 1:**
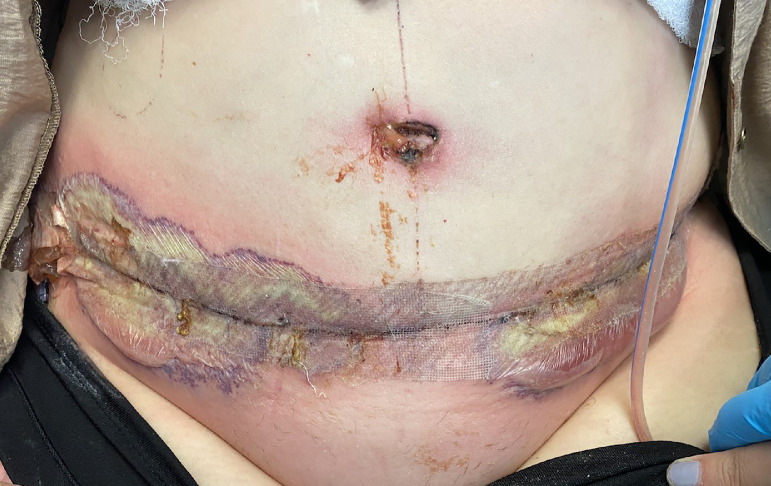
Postoperative day 15. Extensive erythema and ulceration along the transverse abdominal incision with purulent base and violaceous border. There was active purulent drainage from the reconstructed umbilicus.

Our patient is a 51 year-old woman with no prior surgeries and no significant past medical history besides hypothyroidism and vitamin D deficiency who was diagnosed with an invasive breast cancer of her right breast. Patient completed neoadjuvant chemotherapy with paclitaxel and trastuzumab and two months later proceeded with right nipple sparing mastectomy (NSM) and immediate prepectoral breast reconstruction with Mentor® silicone implant placement. She originally wanted to proceed with DIEP flap reconstruction but given the COVID-19 pandemic, a simple outpatient procedure was performed at the time of mastectomy. After two months, she underwent a prophylactic left NSM and bilateral DIEP flap reconstruction. Her body mass index (BMI) at that time was 34.2 kg/m^2^. Total operating time was nine hours. Estimated blood loss was 100 mL. There were no immediate postoperative complications. She had appropriate recovery and was discharged on postoperative day three with oral amoxicillin and clavulanate per protocol. At the time of discharge, her breast and abdominal incisions were healing well, her umbilicus was well perfused and there were no signs or symptoms of infection. On postoperative day (POD) 15, the patient presented to the office with a three day history of abdominal redness, blistering and drainage. She had severe pain around the abdominal incisions. She denied any fever or chills. On physical exam, there was extensive erythema and ulceration along the transverse abdominal incision with a purulent base and violaceous border. There was active purulent drainage from right lateral incision and blistering present on the lateral portion of the transverse abdominal incision on each side. The reconstructed umbilicus was viable but also had ulceration and active purulent drainage (Fig. 1)

**Fig. 2:**
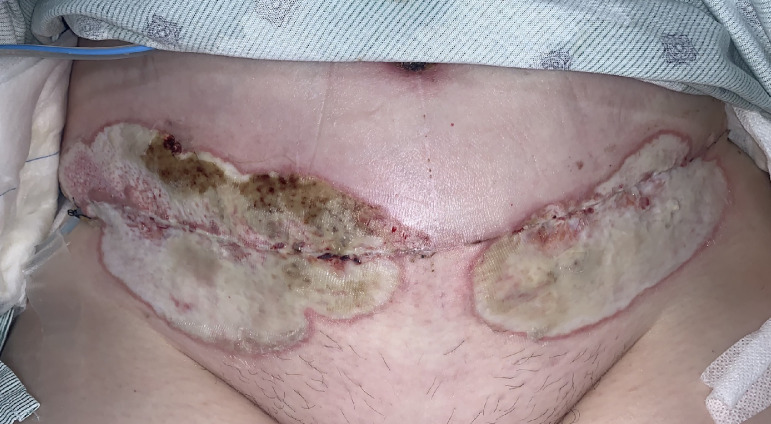
Abdominal wound on day of discharge (POD 24) with mucopurulent base, thin violaceous border and improved erythema.

Due to rapid onset of pain and progression of lesions over the course of 48 h pyoderma gangrenosum was suspected. The patient was admitted for intravenous (IV) antibiotics and started on prednisone 60 mg daily. Laboratory evaluation showed significant leukocytosis of 22,800 per/µL. Initial blood and wound cultures were negative. Dermatology was consulted and recommended to continue steroid therapy and added clobetasol 0.05% topical ointment twice per day on the affected areas. Given the extensive differential and possibility of infection, tissue biopsy and tissue culture were indicated and necessary. Pathergy from biopsy was unlikely given that the patient had stabilized and improved with steroids. Biopsy results showed superficial and deep neutrophilic dermatosis and special stains for microorganisms were negative. The patient continued to have dressing changes with Adaptic™ and abdominal pads twice daily. On POD 21, leukocytosis started improving. By POD 23, the large areas of ulceration along abdominal incision with epidermal sloughing and serous drainage showed marked improvement. The Acute Pain Service was consulted during her hospital course to help manage her pain. The patient was discharged home on POD 24 on oral antibiotics (ciprofloxacin and minocycline) and prednisone 60 mg daily. She remained afebrile throughout her hospital stay. She did not have any debridement performed during her admission given the diagnosis of pyoderma gangrenosum and possible worsening of her wound with any additional surgical trauma (Fig. 2).

**Fig. 3:**
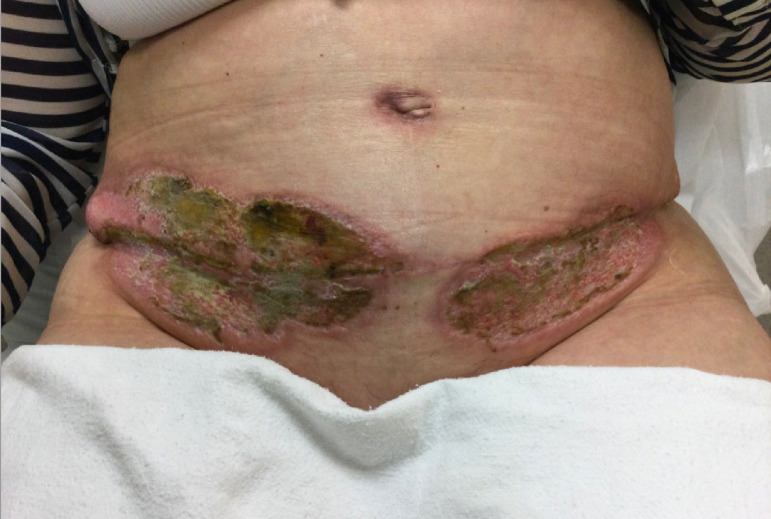
Abdominal wound two months postoperatively. Erythematous shallow ulceration on both sides of the wound with mildly erythematous borders. Umbilicus with mildly erythematous superficial healing scar.

Patient continued with daily application of silvadene cream once daily covered with Adaptic™ and kerlix on her wounds. She continued to follow up weekly and then monthly in the clinic. She finished her antibiotic regimen a couple weeks after discharge. Her steroid therapy was tapered and discontinued after two months (Fig. 3).

**Fig. 4:**
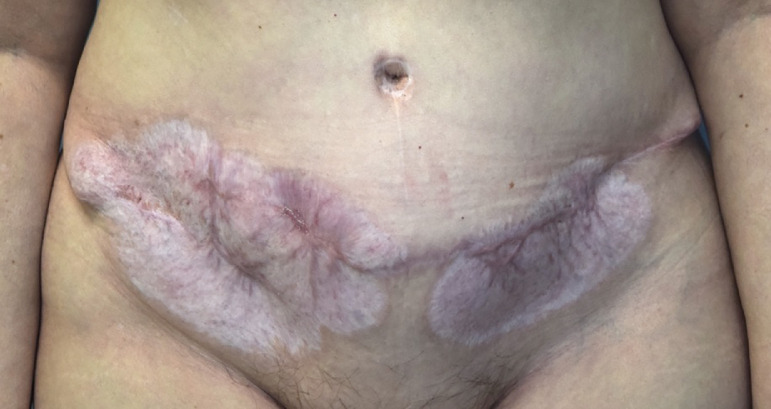
Abdominal wound completely healed 10 months after surgery. Widened scar with no ulceration or surrounding erythema.

At 10 months after her bilateral DIEP flap breast reconstruction, her transverse abdominal incision was well healed, with a widened scar. There was no surrounding erythema or induration and no active drainage present (Fig. 4).

## Discussion

Pyoderma gangrenosum (PG) is a diagnosis of exclusion. Once infection has been ruled out with negative tissue cultures, biopsy can be performed but is not necessary for the diagnosis, which is mainly based on the history and physical exam. Histology demonstrates diffuse neutrophilic dermal inflammation with no evidence of microorganism present. Patients generally have adequate response to steroid therapy. Additional topical treatments can also be offered such as tacrolimus, cyclosporine or 5-aminosalicylic acid.^1^^,^^2^^,^^3^

Although PG most commonly affects the lower extremity, it can also affect the abdomen and the breast. Tuffaha et al. published in 2016 a systematic review of case reports describing PG following different types of breast surgery including autologous breast reconstruction with both breast and donor site being involved.^7^ In 2017, Zelones et al. published a systematic review of PG associated with autologous breast reconstruction, including six deep inferior epigastric perforator (DIEP) flaps. In almost all cases, debridement was performed.^8^ Debridement is not indicated because of the development of pathergy and will exacerbate skin ulceration and lead to disease progression.^5^

Surgeons should keep in mind the diagnosis of PG. Rapid onset and/or progression of ulcerated lesions with significant pain are hallmarks of PG and distinguish it from surgical site infection. Our case demonstrates that when PG is in the clinician's differential diagnosis, prompt treatment can be initiated leading to good outcomes for a difficult problem.

## Ethical approval

Not required

## Funding

None.

## CRediT authorship contribution statement

**Joao Bombardelli:** Conceptualization, Data curation, Formal analysis, Investigation, Writing – original draft. **Janak Parikh:** Conceptualization, Writing – original draft. **Suat Morkuzu:** Supervision, Data curation, Visualization. **Aldona J. Spiegel:** Conceptualization, Funding acquisition, Supervision, Writing – review & editing.

## Declaration of Competing Interest

None.
